# Versatile High-Performance Liquid Chromatography and Ultraviolet Detection-Based Method for the Determination of Thioproline in Pharmaceutical and Cosmetic Products

**DOI:** 10.3390/molecules30153152

**Published:** 2025-07-28

**Authors:** Marta Gaweł, Martyna Płodzik, Rafał Głowacki, Justyna Piechocka

**Affiliations:** 1 Department of Environmental Chemistry, Faculty of Chemistry, University of Lodz, 163/165 Pomorska Str., 90-236 Łódź, Poland; marta.gawel@edu.uni.lodz.pl (M.G.); martyna.plodzik@edu.uni.lodz.pl (M.P.); rafal.glowacki@chemia.uni.lodz.pl (R.G.); 2 Doctoral School of Exact and Natural Sciences, University of Lodz, 12/16 Banacha Str., 90-237 Łódź, Poland

**Keywords:** cosmetics, high performance liquid chromatography, pharmaceutical tablets, timonacic, 2-chloro-1-methylquinolinium tetrafluoroborate, 1,3-thiazolidine-4-carboxylic acid

## Abstract

The article presents the first method based on high-performance liquid chromatography and ultraviolet detection (HPLC-UV) for the determination of timonacic (thioproline, 1,3-thiazolidine-4-carboxylic acid, tPro) in pharmaceutical tablets and face care products (creams, sera, foundations, suncreams). Sample preparation primarily involves solid-liquid extraction (SLE) of tPro with 0.2 mol/L phosphate buffer pH 6, derivatization with 0.25 mol/L 2-chloro-1-methylquinolinium tetrafluoroborate (CMQT), followed by polytetrafluoroethylene (PTFE) membrane filtration. The chromatographic separation of the stable UV-absorbing 2-*S*-quinolinium derivative is achieved within 14 min at 25 °C on a Zorbax SB-C18 (150 × 4.6 mm, 5 µm) column using gradient elution. The eluent consists of 0.1 mol/L trichloroacetic acid (TCA), pH 1.7, in a mixture with acetonitrile (ACN) delivered at a flow rate of 1 mL/min. The analyte is quantified by monitoring at 348 nm. The assay linearity was observed within 0.5–125 μmol/L. The limit of quantification (LOQ) was found to be 0.5 μmol/L. The accuracy ranged from 93.22% to 104.31% and 97.38% to 103.48%, while precision varied from 0.30% to 11.23% and 1.13% to 9.64% for intra- and inter-assay measurements, respectively. The method was successfully applied to commercially available on the Polish market pharmaceutical and cosmetic products.

## 1. Introduction

Thioproline (1,3-thiazolidine-4-carboxylic acid, timonacic, tPro) is a conjugation product of cysteine (Cys) and formaldehyde (FA), which exhibits a wide variety of health-promoting properties. In particular, tPro has attracted interest as a drug due to its antioxidant [1,2], hepatoprotective [3], anticancer [4,5,6,7], and immune-stimulating [8,9,10] properties. In recent years, tPro has also gained an interest in the cosmetics industry. As it is claimed by cosmetics manufacturers, tPro has anti-acne, anti-psoriatic, and anti-aging properties as well as improves structure and stimulates the growth and regeneration of the epidermis, but the information is not supported by the literature. There is a growing number of face care products containing tPro on the global cosmetics market. These findings highlight the need for the development of robust and reliable tPro assays for quality control of such products.

According to Scopus database, merely few methods so far have been developed enabling the determination of tPro in pharmaceutical tablets [11,12,13], urine [14,15,16,17,18,19] and plasma [15] samples collected from people/rats exposed to FA, plasma samples from tPro treated donors [20], serum of lethal prostate cancer patients [21], oxidant-exposed cells (*Escherichia coli*) [22], white and red grape wine [23], traditional Korean fermented foods (soybean paste, soy sauce and red pepper paste) [24], raw and cooked Thai leguminous beans [25], raw and cooked dried Shiitake mushrooms [26] as well as boiled Japanese radish and cod [27]. In addition, proteinic tPro was quantified in proteins obtained from oxidant-exposed *Escherichia coli* [28] and HeLa cells [1]. These methods predominantly exploit separation techniques, such as high-performance liquid chromatography (HPLC) coupled with ultraviolet detection (UV) [20] and (isotope dilution) mass spectrometry (MS) [1,14,15,21,22,23,24,28] as well as gas chromatography coupled with MS [16,19], thermal energy analysis [25,26,27], and nitrogen–phosphorus detector [17,18]. Apart from separation techniques, spectrophotometry [11,13], spectrofluorophotometry [11], and voltammetry [12] were used for the quantification of tPro. These methods primarily involve sample homogenization [11,12,13,25,26,27], deproteinization [20,21], enzymatic digestion [1,28], and cleanup by means of solid-phase [14,15,22], liquid-liquid [15,16,17,18,19,23,25,26,27], solid-liquid (SLE) [11,12,13] extraction, or ultrafiltration [1,28]. In addition, the assays rely on the quantification of tPro or proteinic tPro after derivatization with propyl chloroformate [15], fluorenylmethyloxycarbonyl chloride [1,23,28], acetoxymercuri fluorescein [11], ethyl chloroformate [16,19,22,24], palladium(II) chloride [13], nitrite [25,26,27], and isobutyl chloroformate [17,18], followed by treatment with diazomethane [17,18,25,26,27]. In some cases, derivatization was not necessary to produce a signal of tPro, recorded by HPLC-MS/MS [14,21], HPLC-UV [20], and voltammetric [12] techniques.

None of the above-mentioned methods allows a sensitive and selective determination of tPro using the HPLC-UV technique. There is indeed one HPLC-UV assay for tPro determination, which, along with existing voltammetry and spectrophotometry/spectrofluorophotometry-based methods enabling the quantification of tPro in pharmaceutical tablets [11,12,13], suffers from poor selectivity (tPro signal has been recorded at 205 nm). In addition, tPro has not been quantified in cosmetic products. Thus, the present paper aims to provide a versatile HPLC-UV based method for the determination of tPro in pharmaceutical tablets and face care products. Such an approach will make it possible to widely use the delivered assay in worldwide quality control laboratories.

## 2. Results and Discussion

The article presents the first HPLC-UV assay for the determination of tPro in pharmaceutical and cosmetic products. The following (sub)sections of the report provide essential data on the development, validation, and in-study use of the method described herein. Experiments were performed using a model sample containing 0.1 mmol/L tPro, powdered tablets, and face care products. The default mass of the solid sample equaled 0.003 g and 0.3 g for tablets and cosmetics, respectively. Regardless of the type of sample, the same observations were made. The results are presented in the order in which the experiments were conducted.

### 2.1. Sample Preparation

Sample preparation primarily involves (1) SLE of tPro from pharmaceutical/cosmetic product with 0.2 mol/L phosphate buffer (PBS) pH 6; (2) derivatization of the analyte with 2-chloro-1-methylquinolinium tetrafluoroborate (CMQT), followed by polytetrafluoroethylene (PTFE) membrane filtration of the resulting solution. Then, tPro in the form of 2-*S*-quinolinium derivative (tPro-CMQT) is separated and quantified by HPLC-UV assay.

#### 2.1.1. Extraction

In order to extract and separate tPro from the solid sample components, samples were processed using SLE. During the process, samples were placed in a vortex mixer at ambient temperature to improve batch-to-batch reproducibility. Firstly, several miscible solvents in water were tested, including acetonitrile (ACN), methanol (MeOH), ethanol, 2-propanol, acetone, tetrahydrofuran, dimethylformamide, dimethyl sulfoxide, dioxane, 0.2 mol/L PBS pH 6, and water itself. Among them, the application of 0.2 mol/L PBS pH 6 resulted in the highest SLE efficiency (Figure S1a) in contrast with other tPro assays in which powdered tablets were sonicated with hot water [12,13] or MeOH [11]. Then, other parameters that could promote the SLE efficiency were evaluated, namely the amount of acceptor phase, agitation frequency, and time. The volume of 0.2 mol/L PBS pH 6, tested in the range of 500–2000 µL, had a negligible impact on it (Figure S1b). The progressive rise of the signal peak area and improvement of the assay reproducibility were observed in parallel with the rise in shaking frequency from 300 rpm to 1500 rpm without further changes up to the highest possible shaking frequency of 3000 rpm (Figure S1c). The extraction process was completed in 5 min when samples were mixed with 1000 µL of 0.2 mol/L PBS pH 6 and placed in a vortex mixer set to 2000 rpm (Figure S1d). Taking into consideration relatively high concentrations of tPro in study samples, sample dilution was not a problem. After centrifugation, 10 µL of the supernatant was subjected to derivatization.

#### 2.1.2. Derivatization

**Figure 1:**
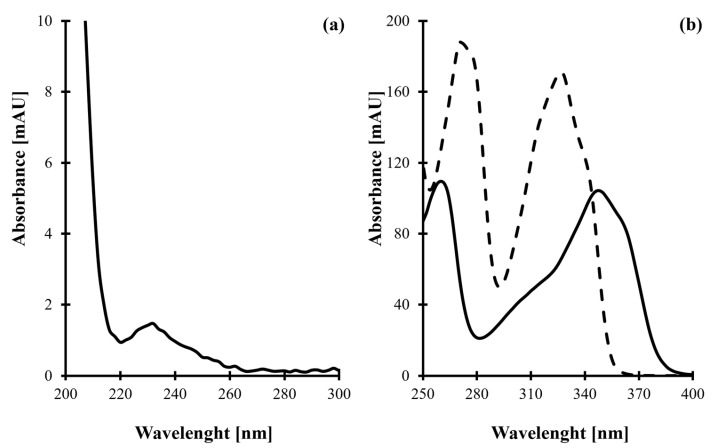
(**a**) UV absorption spectrum of tPro (100 µmol/L) obtained by analyzing its standard solution prepared in water, assayed using a UV-1900 spectrophotometer. (**b**) UV absorption spectra of tPro-CMQT derivative (solid line, 100 µmol/L) and CMQT (dashed line, 15 mmol/L) obtained by analyzing quality control sample assayed according to procedures described in Section 3.5 and Section 3.6.

Despite that tPro is a UV-absorbing molecule (Figure 1a), its poor UV detectability and chromatographic behavior in reversed phase (RP) HPLC mode of separation due to high polarity of the molecule have generated a need for sample derivatization. For this purpose, CMQT was used. Thus far, CMQT has been documented to be an effective and thiol-specific derivatizing reagent when determining low- [29,30,31,32,33,34,35,36,37,38,39,40] and high-molecular-mass [36] sulfur-containing compounds by HPLC [29,30,31,32,35,36,39,40] and capillary electrophoresis [29,33,34,37,38] coupled with UV [29,30,32,33,34,35,36,37,38,39,40] or MS [31] detection. More importantly, Piechocka et al. have recently demonstrated that CMQT is a suitable derivatizing agent for 2-(3-hydroxy-5-phosphonooxymethyl-2-methyl-4-pyridyl)-1,3-thiazolidine-4-carboxylic acid (HPPTCA) [32]. It has been shown that a stable 2-*S*-quinolinium derivative of HPPTCA, exhibiting an absorption peak near 355 nm, is formed under acidic conditions (0–1 pH range) in 5 h at room temperature.

**Figure 2:**
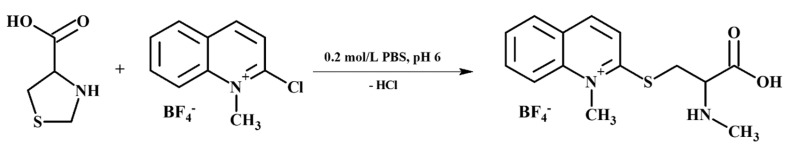
Schematic representation of the chemical derivatization reaction of tPro with CMQT affording the 2-S-quinolinium derivative of tPro.

Firstly, it has been found that tPro, like HPPTCA, reacts with CMQT, resulting in tPro-CMQT. The reaction is based on simultaneous thiazolidine ring opening by C-S bond breaking and nucleophilic substitution of the chlorine atom in the quinolinium ring by the released free thiol group of tPro (Figure 2) [32]. It produces UV-absorbing tPro-CMQT and is accompanied by a bathochromic shift of absorption bands of the derivative compared with its precursors (Figure 1b). It is also worth noting that derivatization helped to increase the UV detectability of the analyte and the sensitivity of the method. In short, the extinction coefficient of tPro-CMQT is about 50 times higher than the extinction coefficient of tPro, as shown in Figure 1. In addition, upon derivatization, tPro was converted to more hydrophobic tPro-CMQT that could be effectively retained and separated in the RP-HPLC mode of separation. Without derivatization, tPro cannot be retained in RP-HPLC.

Several parameters that may have an impact on chemical derivatization efficiency were tested, including pH of reaction medium, quantity of CMQT, temperature, and reaction time. The derivatization was conducted in 0.2 mol/L PBS as previously reported [29,41,42,43]. It has been recognized that the rate and efficiency of reaction were dependent on pH, studied within the range of 4.2–11.0 (Figures S2 and S3a). In general, the higher the pH, the faster the reaction is equilibrated, while the application of 0.2 mol/L PBS at pH 6 resulted in the highest process efficiency (Figure S3a). Interestingly, tPro-CMQT was not formed under either extremely acidic and alkaline conditions (pH < 4.2 and pH > 10). The progressive rise of the tPro-CMQT peak area was observed concomitant with the rise in concentration of CMQT in the reaction mixture from 0.1 to 10 mmol/L, and then the tPro-delivered signal remained without changes up to 50 mmol/L (Figure S3b). Moreover, the reduction of reaction time from 60 to 15 min was observed in parallel with temperature rise from 20 to 50 °C (Figure S3c), with no impact on the high yields of the derivatization process. For routine analysis, 10 µL of the extract was diluted with 930 µL of 0.2 mol/L PBS, pH 6, and 60 µL of 0.25 mol/L CMQT. To reduce energy consumption and simplify the sample pretreatment procedure, the reaction was conducted at room temperature for at least 60 min (Figure S3d). By contrast, low- [29,30,31,32,33,34,35,36,37,38,39,40] or even high-molecular-mass [36] sulfur-containing compounds require less than 30 min to efficiently react with CMQT, with the exception of HPPTCA that needs at least 5 h for completion of the derivatization reaction [32].

#### 2.1.3. Filtration

Samples were purified by passing through syringe filters prior to HPLC analysis. High recovery nylon, polyethersulfone, and PTFE-based membrane syringe filters, from different manufacturers, with nominal porosity of 0.20–0.45 µm, were tested. Regarding tPro-CMQT, membrane type has had a negligible effect on its recovery, reaching 69.29–99.59% (Figure S4). Processed samples were thus clarified using PTFE syringe filters, providing the recovery of tPro-CMQT of 99.59%, regardless of its concentration.

#### 2.1.4. Stability of Processed Samples

As described in the literature, 2-*S*-quinolinium derivatives of HPPTCA and some low molecular mass sulfur-containing compounds are stable under acidic conditions [29,30,31,32,33,34,35,36,37,38,39,40]. In order to prolong the stability of the reaction product and terminate the reaction, samples are usually acidified with 3 mol/L perchloric acid (PCA) [29,30,32,33,34,35,37,38,39,40]. Regarding tPro-CMQT, the derivative was found to be stable at 25 °C in both 0.2 mol/L PBS, pH 6, and samples treated with 3 mol/L PCA (Figure S5), excluding the need for sample acidification.

The stability of tPro-CMQT in samples was tested at 25 °C and 4 °C [44]. It has been demonstrated that processed samples remain stable for at least 1 week, regardless of the examined concentration of the analyte and conditions. The tPro-CMQT recovery of 99.18% and 99.80% was observed when study samples were stored at 25 °C and 4 °C, respectively (Figure S6a,b). The freeze-thaw stability testing of such samples has indicated that the analysis of even five times thawed samples can provide meaningful results (Figure S7) since no more than 4.49% of the analyte was decomposed. The obtained results are in agreement with the literature data and support information that 2-*S*-quinolinium derivatives of HPPTCA and some low/high molecular mass sulfur-containing compounds are stable [29,30,31,32,33,34,35,36,37,38,39,40]. Regarding tPro-CMQT, this is the first piece of data on this topic.

### 2.2. Chromatographic and Detection Conditions

The assay relies on the separation of tPro-CMQT using RP-HPLC mode of separation and UV detection.

#### 2.2.1. Selection of Separation Conditions

**Figure 3:**
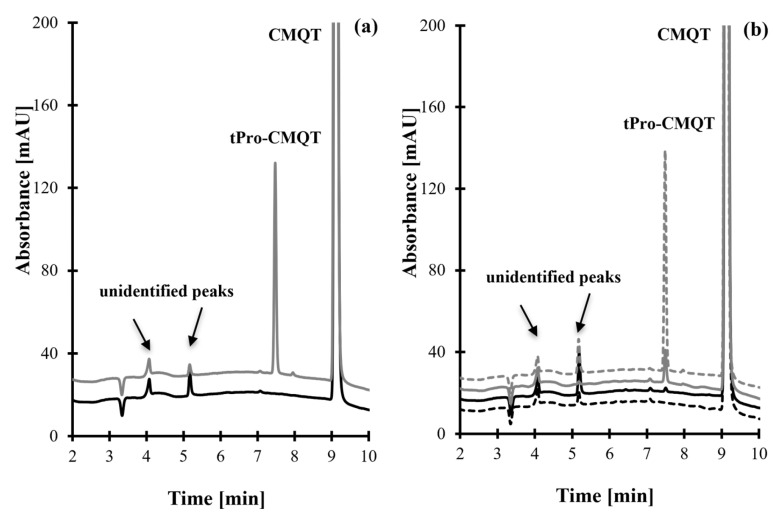
Representative chromatograms of standard solutions, pharmaceutical tablets, and face care products prepared according to the procedure described in Section 3.5. Chromatographic conditions were as described in Section 3.6. (**a**) Blank standard solution (black line) and standard solution of tPro (100 µmol/L, grey line). (**b**) Pharmaceutical tablet (grey dashed line), face cream (grey line), face foundation (black dashed line), and face serum (black line). The analyte-delivered signal tPro-CMQT appears at 7.50 min.

So far, it has been demonstrated that ion pair RP-HPLC coupled with UV detection is suitable for determining low and high-molecular-mass sulfur-containing compounds after derivatization with CMQT [29,41,42,43]. In this study, samples were analyzed according to a previously published method by Piechocka et al. [32], enabling an efficient separation of plasma HPPTCA, glutathione (Glu), homocysteine (Hcy), Cys, and cysteinyl-glycine (Cys-Gly). Under fixed conditions, efficient resolution of tPro-CMQT from other study sample components was achieved. The analyte yielded one chromatographic peak, which was eluted within 7.50 min and easy to differentiate from the other responses on the chromatogram (Figure 3a,b and Figure S8a,b). As satisfactory method’s performance was achieved, HPLC conditions were not optimized during this study. The analysis time of 14 min is longer than in other tPro assays, such as HPLC-MS/MS [14,15,22,24], where it is 8–12 min. In other cases, the duration of HPLC analysis is more favorable than in other HPLC-UV [20], HPLC-MS/MS [1,21,23,28], and GC-MS [16,17,18,19,25,26,27] based methods, where it is 19–38 min. In relation to the tPro methods based on spectrophotometry [11,13], spectrofluorophotometry [11], and voltammetry [12], the analysis time is difficult to specify, and no information on this topic can be found in the original articles.

#### 2.2.2. Selection of Detection Conditions

During this study, it has been demonstrated that tPro is converted into its 2-*S*-quinolinium derivative upon reaction with CMQT. It has also been found that the derivatization reaction produces a UV-absorbing tPro-CMQT derivative, exhibiting a well-defined absorption peak near 348 nm (Figure 1b), similarly to other CMQT derivatives [29,30,31,32,33,34,35,36,37,38,39,40]. The analyte was thus determined using 348 nm to increase the sensitivity of the method. In order to confirm the identity of the 7.50 min peak as derived from tPro present in real samples, the retention time and UV spectrum were compared with the corresponding set of data obtained by analyzing an analytical standard of tPro.

### 2.3. Validation of the Method

Full validation of the HPLC-UV assay was performed taking into account the latest International Council for Harmonization guideline for bioanalytical methods validation [44]. The same protocol was employed as described in detail in our previously published articles [32,45]. The description of the validation process is also provided as part of the Supplementary Materials. Parameters, such as system suitability, selectivity, linearity, intra-/inter-assay precision and accuracy, limit of quantification (LOQ), carry-over, matrix effect, reinjection reproducibility, and recovery (extraction efficiency) were evaluated.

#### 2.3.1. System Suitability

System suitability testing included checking retention time, peak area, symmetry factor, and theoretical plate number. The coefficient of variation (CV) equaled 0.04% and 0.39% regarding retention time and peak area, respectively (acceptance criteria ≤1%). The mean symmetry factor was 0.92 (acceptance criteria 0.8–1.5), and the theoretical plate number was 71,368 (acceptance criteria ≥2000) for low, middle, and high quality control samples assayed by HPLC-UV method in ten replicate injections.

#### 2.3.2. Selectivity

Selectivity studies evaluated interferences originating from any UV-absorbing components of study samples as well as HPPTCA and some low molecular mass sulfur-containing compounds, which are known to react with CMQT [29,30,31,32,33,34,35,36,37,38,39,40]. The selectivity was assessed using (1) blank standard solution, (2) standard solution of HPPTCA and reduced forms of Cys, Glu, Hcy, Cys-Gly, N-acetyl-cysteine, captopril, mesna, cysteamine, tiopronin, methimazole, and γ-glutamyl-cysteine, (3) pharmaceutical tablets and face care products (creams/foundations/sera) without tPro from six sources, and (4) pharmaceutical and cosmetic products containing tPro. As shown in Figure 3 and Figure S8, no response attributable to interfering components was observed at the retention time of tPro-CMQT in blank samples. Additionally, the results from tPro-CMQT delivered peak purity testing confirmed the selectivity of the HPLC-UV assay toward tPro.

#### 2.3.3. Linearity

An external standard addition method was employed to examine the calibration range. Calibration curves consisted of blank sample and nine calibrators at the level of 0.5, 1, 5, 10, 25, 50, 75, 100, 125 µmol/L. They were run in triplicate over 3 days. To describe the concentration-response (tPro-CMQT peak area) relationship, a least-squares regression model was used. The calibration standards fitted well into the linear regime (correlation coefficient values equal to at least 0.9999). Intra- and inter-assay changes in the slope of regression lines were not observed. The CV of slope of regression lines equaled 1.31% and 1.67% for intra- and inter-assay measurements, respectively (Table 1).

#### 2.3.4. Precision and Accuracy

Along with linearity evaluation, the precision and accuracy of the method were assessed. The precision was expressed as the CV of measurement repeatability. Accuracy was calculated by expressing the mean measured amount as a percentage of the added amount of tPro (nominal concentration). The accuracy was within 93.22–104.31% and 97.38–103.48% for intra- and inter-assay measurements, respectively. The precision varied from 0.30% to 11.23% and 1.13% to 9.64% for intra- and inter-assay variations, respectively (Table 2).

#### 2.3.5. The Limit of Quantification

The LOQ was assessed using the standard deviation method. The LOQ was calculated using the equation LOQ = 10 × SD_a_/b, where SD_a_ is the standard deviation of the response and b is the slope of the regression line. Calculations were performed using parameters of calibration curves performed for pharmaceutical tablets and face care products (creams/foundations/sera), which did not contain tPro, and 0.2 mol/L PBS, pH 6. SD_a_ of the instrument’s response was calculated based on data obtained from the analysis of a set of ten standard solutions of tPro (0.5 µmol/L), a concentration nominated as LOQ using the signal-to-noise method. Throughout this study, it was confirmed that the LOQ equals 0.5 µmol/L (66.60 µg/L) as this concentration of tPro produces a detector response with precision that did not exceed 11.23% and accuracy in the range of 93.85–101.51%. In relation to other tPro methods enabling the determination of tPro in pharmaceutical tablets, the LOQ equaled 28.48 mg/L [13], 43.50 µg/L [12], 0.18 mg/L [11], and 0.55 µg/L [11] when spectrophotometry [11,13], spectrofluorophotometry [11], and voltammetry [12] were employed.

#### 2.3.6. Carry-Over Effect

In order to test the carry-over effect, a blank standard solution sample was analyzed after the calibrator at the upper LOQ. No response attributable to tPro-CMQT was registered in the blank samples, indicating that the carry-over effect does not occur.

#### 2.3.7. Matrix Effect

The matrix effect evaluation relied on the comparison of calibration curves of six independent sources of pharmaceutical tablets and face care products (creams/foundations/sera), which did not contain tPro, against a calibration curve of surrogate matrix (0.2 mol/L PBS, pH 6). The CV of the slope of regression lines equaled 12.91% indicating that the obtained results are not affected by matrix effect.

#### 2.3.8. Reinjection Reproducibility

Low, middle, and high quality control samples were analyzed in ten replicate injections. The CV value of tPro-CMQT peak area equaled 0.39% indicating that study samples can be reinjected at least 10 times.

#### 2.3.9. Extraction Efficiency (Recovery)

Recovery was determined by comparing the analyte response in a real sample that was spiked with the target compound and processed, with the response in the same sample that was subjected to SLE and then spiked with tPro. Recovery experiments were performed by comparing the analytical results for extracted samples that referred to pharmaceutical tablets and face care products (creams/foundations/sera) from multiple sources, containing known amounts of the analyte at three concentrations (low, medium, and high). Samples were run in triplicate. All concentrations were tested with the use of calibration curves prepared on that occasion. Extraction efficiency was calculated by expressing the mean measured amount of the analyte extracted as a percentage of the total (nominal) amount of tPro in the sample, considering its initial composition and added amount of tPro. Importantly, the recovery was reproducible regardless of tPro concentration and product type. The recovery of tPro from real samples was within 99.35–104.12% and 99.50–103.07% in relation to pharmaceutical tablets and cosmetics, respectively. Such high extraction efficiency ensures that the measured concentration of tPro accurately reflects its true concentration in the study samples.

### 2.4. Application of the Method

The HPLC-UV method was used to determine tPro in pharmaceutical and cosmetic products containing tPro as declared by the manufacturer. An external standard addition method was employed to quantify the content of tPro in study samples. The content of tPro was calculated using the regression equation of the calibration line generated on that occasion. The analyte was present in almost all studied samples (Table 3). The estimated content of tPro in tablets agreed with that declared by the manufacturer, indicating the validity of the method. The tPro content of cosmetic products was not provided by the producer.

## 3. Materials and Methods

### 3.1. Reagents and Materials

In general, commercially available chemicals and at least of analytical reagent grade were used. Sodium phosphate dibasic heptahydrate (≥98%), sodium phosphate monobasic monohydrate (≥99.0%), tPro (98%), HPLC gradient grade ACN (≥99.9%), trichloroacetic acid (TCA) (≥99.0%) were from Sigma–Aldrich (St. Louis, MO, USA). Sodium hydroxide (98.8%) was from J.T. Baker (Deventer, The Netherlands). CMQT (≥95%) was prepared in our laboratory as previously described [46]. Ultrapure deionized water was produced in our laboratory. Samples were filtered prior to HPLC analysis using syringe filters with 0.20 μm PTFE membrane (Agilent Technologies, Waldbronn, Germany).

### 3.2. Instrumentation

An Agilent 1220 Infinity HPLC system equipped with a binary pump integrated with a two-channel vacuum degasser, autosampler, temperature-controlled column compartment, and UV detector (Agilent Technologies, Waldbronn, Germany) was used. Instrument control, data acquisition, and analysis were performed using the OpenLAB CDS software, version A.01.05. The analyte was separated on a ZORBAX SB-C18 (150 × 4.6 mm, 5.0 μm) column from Agilent Technologies (Waldbronn, Germany).

For sample shaking, the Multi-Speed Vortex MSV-3500 (Biosan, Riga, Latvia) was used. During this study, a Mikro 220R centrifuge with fast cool function (Hettich Zentrifugen, Tuttlingen, Germany) and a FiveEasy F-20 pH-meter (Mettler Toledo, Greifensee, Switzerland) were also used. For absorbance measurements, the UV-1900 spectrophotometer with the UVProbe software, version 2.70 (Shimadzu, Kyoto, Japan) was employed. Water was purified using a Direct-Q 3 UV water purification system (Millipore, Vienna, Austria).

### 3.3. Stock Solutions

All solutions were prepared weekly with the use of ultrapure deionized water.

The stock solutions of 0.1 mol/L tPro and 0.25 mol/L CMQT were prepared by dissolving an appropriate amount of solid substance in water. The solutions were kept at 4 °C without noticeable change in their content (Figure S9a,b). They were stored in polypropylene (PP) microcentrifuge tubes. The working solutions of tPro were prepared daily by diluting a standard solution with water as needed and processed without delay.

The stock solution of 0.2 mol/L PBS at pH 6.0 was prepared by dissolving an appropriate amount of phosphate dibasic in water and then adjusting the pH with an appropriate volume of 0.2 mol/L water solution of sodium phosphate monobasic. The PBS was stored in a tight glass bottle at room temperature.

The mobile phase component used in HPLC-UV experiments, consisting of 0.1 mol/L TCA at pH 1.7, was prepared by dissolving an appropriate amount of the solid substance in water and adjusting the pH with an appropriate volume of sodium hydroxide (1 mol/L) by potentiometric titration. The solution was stored in tight amber glass bottle at room temperature. Sodium hydroxide (1 mol/L) was prepared by dissolving an appropriate amount of base in water. The solution was stored in tight glass flasks at ambient temperature.

### 3.4. Sample Collection

Pharmaceutical and cosmetic products were obtained from local drugstores and pharmacies. All products were commercially available on the Polish market, manufactured and purchased in 2025. They included pharmaceutical tablets Heparegen 100 mg (Bausch Health Ireland Limited, Dublin, Ireland) and Timohep 100 mg (Solinea Sp. z o.o., Ciecierzyn, Poland) as well as face care products such as creams, serum, foundation, and suncream manufactured by Dr Irena Eris S.A. The products were stored as recommended by the manufacturer until analysis.

### 3.5. Pharmaceutical and Cosmetic Products Preparation

**Figure 4:**
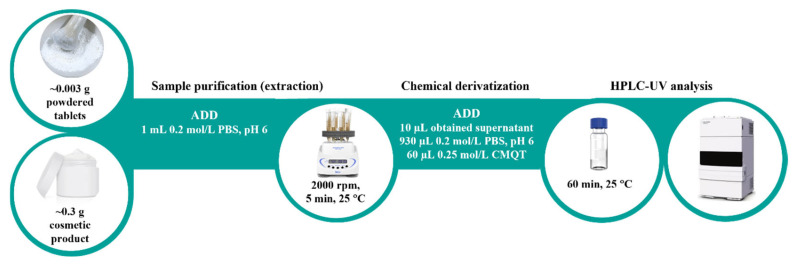
The experimental procedure for tPro determination in pharmaceutical and cosmetic products described in Section 3.5.

A powdered pharmaceutical tablet (~0.003 g) or face care product (~0.3 g) was mixed with 1 mL of 0.2 mol/L PBS at pH 6.0 in a 2 mL PP microtube. In the next step, the analyte was extracted by vortexing at 2000 rpm for 5 min at 25 °C. Then, the resulting mixture was centrifuged (optional, 12,000× *g*, 25 °C, 5 min), and the supernatant (10 μL) was transferred into a 1st hydrolytic class glass HPLC vial containing 930 μL of 0.2 mol/L PBS at pH 6.0 and 60 μL of 0.25 mol/L CMQT. The reaction mixture was put aside for 60 min at room temperature. A 5 µL aliquot of the resulting solution was introduced into the HPLC-UV system (Figure 4).

### 3.6. Chromatographic Analysis Conditions

Samples were analyzed according to our previously published method [32]. The chromatographic separations were performed on ZORBAX SB-C18 (150 × 4.6 mm, 5.0 μm) column at room temperature, using linear gradient elution (0–8 min, 11–40% B; 8–12 min, 40–11% B) with the mobile phase consisted of 0.1 mol/l TCA pH 1.7 (solvent A) and ACN (solvent B), delivered at the flow rate of 1 mL/min. The column was re-equilibrated between analyses by setting the post-run conditioning at 11% B for 2 min. The effluent was monitored with a UV detector at 348 nm with a bandwidth of 4 nm using 390 nm ± 20 nm as a reference wavelength. The detector was set to collect data within 2–10 min.

## 4. Conclusions

To the best of our knowledge, the article presents the first HPLC-UV assay for the determination of tPro in commercially available pharmaceutical tablets and face care products (creams, serum, foundation, suncream). The research also confirms our previously published data indicating that CMQT is a suitable derivatizing agent for 1,3-thiazolidine-4-carboxylic acids [32]. Regarding tPro, this is the first piece of data on this topic. Excellent stability of tPro-CMQT in study samples represents a considerable advantage along with a simple sample preparation procedure, possibility of handling a set of twenty-four samples in only 70 min, as well as no need to work under a time regime to obtain meaningful results (Figure 4). The process consumes approximately 2 mL of inexpensive and non-toxic chemicals per sample. In relation to other tPro assays, pharmaceutical tablets sample preparation time was indeed shorter (15–20 min), but the process consumes more chemicals (35–110 mL) [11,12,13]. Overall, the use of common equipment in worldwide laboratories and the high performance of the presented method allow its use in quality control testing of pharmaceutical and cosmetics products for tPro.

## Figures and Tables

**Table 1:** Validation data corresponding to intra-assay measurements (n = 3).

Regression Equation	R	CV Slope (%)	Range (µmol/L)	Precision (%)		Accuracy (%)		LOQ (µmol/L)
				Min	Max	Min	Max	
y = 4.0506x + 0.3992	0.9999	1.31	0.5–125	0.30	11.23	93.22	104.31	0.5

Abbreviations: CV, coefficient of variation; LOQ, limit of quantification; R, correlation coefficient.

**Table 2:** Precision and accuracy of the data from 3 days of measurements (n = 3).

Concentration (µmol/L)	Precision (%)		Accuracy (%)	
	Intra-Assay	Inter-Assay	Intra-Assay	Inter-Assay
0.5	11.23	9.64	93.85	101.51
10	3.14	4.17	104.31	103.48
50	1.47	1.75	99.89	100.80
100	0.77	2.00	100.53	101.40

**Table 3:** The estimated tPro content in pharmaceutical and cosmetic products, expressed as a mean value of the measured amount.

Product Type	No.	tPro Content of Product		
		Estimated		Declared by Producer
		(µg ± SD/g Product)	(mg ± SD/Tablet)	
Tablet 1	1	-	99.1 ± 2.1	100 mg/tablet
Tablet 2	2	-	99.8 ± 4.1	100 mg/tablet
Face cream 1	3	16.3 ± 0.3	-	No data
Face cream 2	4	52.2 ± 2.1	-	No data
Face cream 3	5	159.8 ± 15.3	-	No data
Face cream 4	6	16.5 ± 1.6	-	No data
Face cream 5	7	6.9 ± 0.7	-	No data
Face cream 6	8	8.6 ± 1.1	-	No data
Face cream 7	9	5.1 ± 0.5 *	-	No data
Face cream 8	10	7.2 ± 0.5	-	No data
Face cream 9	11	205.6 ± 14.6	-	No data
Face cream 10	12	7.7 ± 1.2	-	No data
Face cream 11	13	Not found	-	No data
Face cream 12	14	Not found	-	No data
Face serum	15	0.1 ± 0.0 *	-	No data
Face foundation	16	Not found	-	No data
Face foundation	17	Not found	-	No data
Suncream	18	14.5 ± 1.6	-	No data

Abbreviations: SD, standard deviation; tPro, thioproline. Legend: (*) estimated concentration below LLOQ.

## Data Availability

Essential data are contained within the article and the Supplementary Materials file. In addition, the dataset generated and analyzed during this study, which contributed to the article, can be made available by the corresponding author upon reasonable request, provided that the request does not compromise intellectual property interests. Samples are not available from the authors.

## Supplementary Materials

The following supporting information can be downloaded at https://www.mdpi.com/article/10.3390/molecules30153152/s1: Figure S1. Extraction efficiency as a function of (a) type of extraction solvent, (b) 0.2 mol/L PBS pH 6 volume, (c) shaking frequency, and (d) time, expressed as a peak area of tPro-CMQT. Figure S2. Reactivity of tPro toward CMQT in 0.2 mol/L PBS at pH 4.2 (dots), 6.0 (triangles) and 9.0 (squares) at room temperature as a function of time, expressed as a peak area of tPro-CMQT. Figure S3. Derivatization reaction efficiency as a function of (a) pH of reaction medium, (b) concentration of CMQT in reaction mixture, (c) temperature, (d) time, expressed as a peak area of tPro-CMQT. Figure S4. Membrane filtration efficiency of tPro-CMQT as a function of the type of membrane filter, expressed as a percentage of tPro-CMQT recovery. Figure S5. Examination of tPro-CMQT stability at room temperature in 0.2 mol/L PB pH 6 (dots) and samples treated with 3 mol/L PCA (triangles) as a function of time, expressed as a peak area of tPro-CMQT. Figure S6. Examination of tPro-CMQT stability at (a) room temperature and (b) 4 °C as a function of time, expressed as the percentage of the tPro-CMQT recovery. Figure S7. Freeze-thaw stability of tPro-CMQT in processed samples, expressed as a peak area of tPro-CMQT. Figure S8. Representative chromatograms of standard solutions prepared according to the procedure described in Section 3.5. Chromatographic conditions were as described in Section 3.6. (a) Standard solution of tPro (black line) (100 µmol/L) and standard solution of HPPTCA, Cys, Glu, Hcy, Cys-Gly, N-acetyl-cysteine, captopril, mesna, cysteamine, tiopronin, methimazole, and γ-glutamyl-cysteine (grey line) (100 µmol/L). Under these conditions, their corresponding 2-*S*-quinolinium derivatives elute in the following order: 1-mesna, 2-HPPTCA, 3-N-acetyl-cysteine, 4-Glu, 5-γ-glutamyl-cysteine, 6-tiopronin, 7-methimazole, 8-Hcy, 9-cysteamine, 10-captopril, 11-Cys, 12-Cys-Gly, 13-CMQT. (b) Pharmaceutical tablet (black solid line), face cream (grey line), face foundation (black dashed line) and face serum (grey dashed line) without tPro. Figure S9. Examination of stability of stock solution of tPro at (a) room temperature and (b) 4 °C as a function of time, expressed as a peak area of tPro-CMQT.

## Abbreviations

The following abbreviations are used in this manuscript:

ACN	Acetonitrile
CMQT	2-Chloro-1-methylquinolinium tetrafluoroborate
Cys	Cysteine
Cys-Gly	Cysteinyl-glycine
CV	Coefficient of variation
FA	Formaldehyde
Glu	Glutathione
Hcy	Homocysteine
HPLC	High performance liquid chromatography
HPPTCA	2-(3-hydroxy-5-phosphonooxymethyl-2-methyl-4-pyridyl)-1,3-thiazolidine-4-carboxylic acid
LOQ	Limit of quantification
MeOH	Methanol
MS	Mass spectrometry
PBS	Phosphate buffer
PCA	Perchloric acid
PP	Polypropylene
PTFE	Polytetrafluoroethylene
RP	Reversed phase
SLE	Solid-liquid extraction
TCA	Trichloroacetic acid
tPro	Thioproline
tPro-CMQT	2-S-quinolinium derivative of thioproline
UV	Ultraviolet detection
