# Supplementary material for: Versatile High-Performance Liquid Chromatography and Ultraviolet Detection-Based Method for the Determination of Thioproline in Pharmaceutical and Cosmetic Products
Source: Molecules. 2025 Jul 28;30(15):3152. doi: 10.3390/molecules30153152 (PMC12348203; doi:10.3390/molecules30153152)
Supplement: Supplementary file 1 [file molecules-30-03152-s001.zip › molecules-3736336-supplementary.pdf]

Versatile high performance liquid chromatography and ultraviolet detection based method for the determination of thioproline in pharmaceutical and cosmetic products

Marta Gaweł[1,2], Martyna Płodzik[1], Rafał Głowacki[1], Justyna Piechocka[1]*

[1]*University of Lodz, Faculty of Chemistry, Department of Environmental Chemistry, 163/165 Pomorska Str., 90-236 Łódź, Poland*

[2]*University of Lodz, Doctoral School of Exact and Natural Sciences, 12/16 Banacha Str., 90-237 Łódź, Poland*

ORCID iD: 0009-0006-2241-6877 (M.G.), 0000-0003-0071-1470 (R.G.), 0000-0002-1160-3160 (J.P.)

marta.gawel@edu.uni.lodz.pl (M.G.), martyna.plodzik@edu.uni.lodz.pl (M.P), rafal.glowacki@chemia.uni.lodz.pl (R.G.), justyna.piechocka@chemia.uni.lodz.pl (J.P.)

Corresponding author

* Justyna Piechocka, University of Lodz, Faculty of Chemistry, Department of Environmental Chemistry, 163/165 Pomorska Str., 90-236 Łódź, Poland

e-mail: justyna.piechocka@chemia.uni.lodz.pl

Tel.:+48-42-635 5844

Fax: +48-42-635 5832

Supplementary results produced during development of the presented HPLC-UV assay

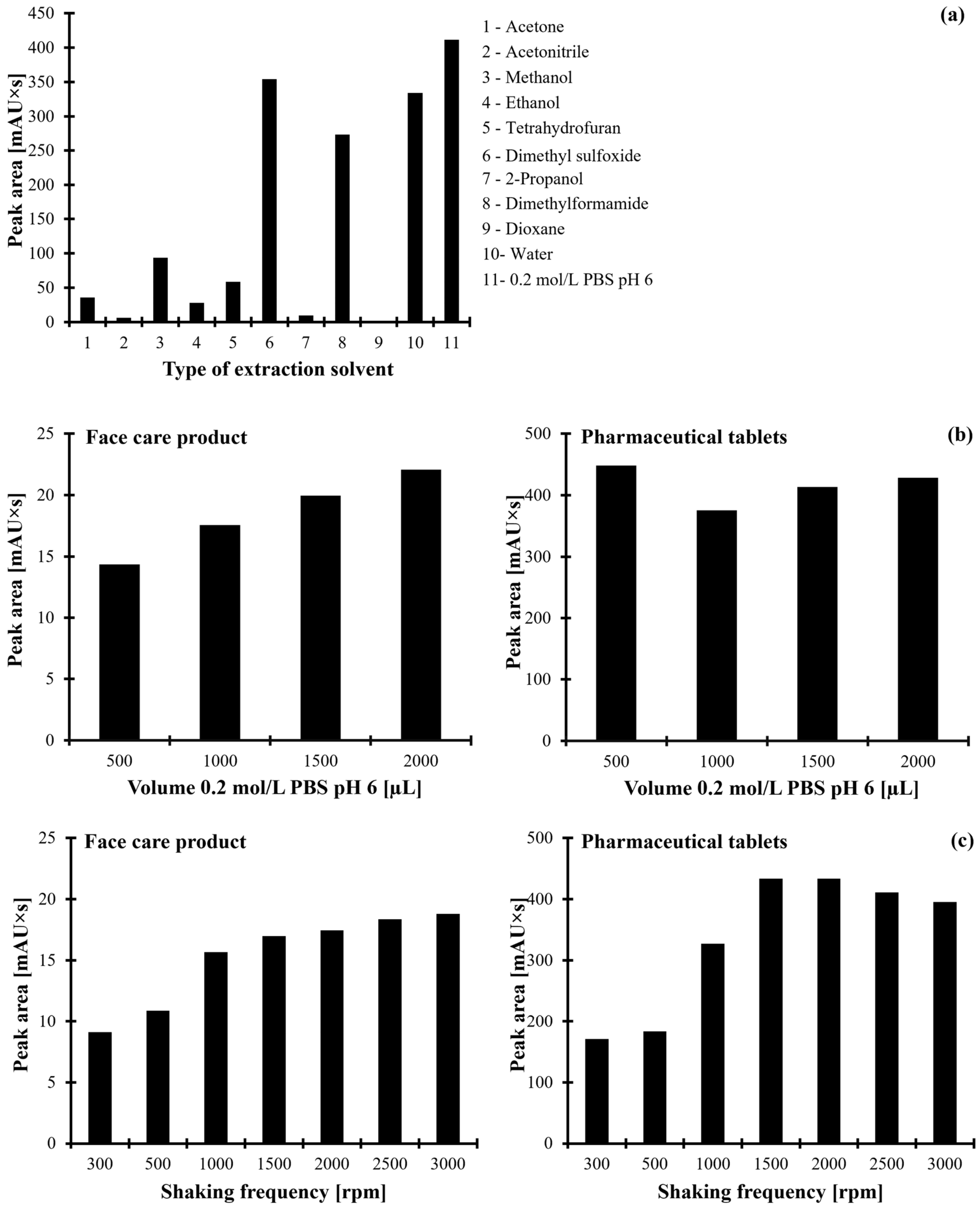

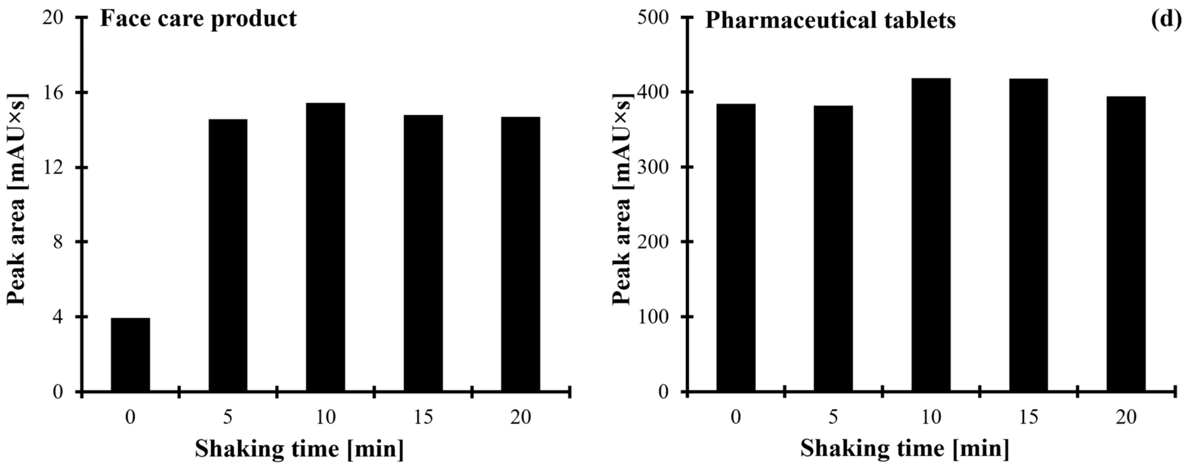

Figure S1. Extraction efficiency as a function of (a) type of extraction solvent, (b) 0.2 mol/L PBS pH 6 volume, (c) shaking frequency, and (d) time, expressed as a peak area of tPro-CMQT.

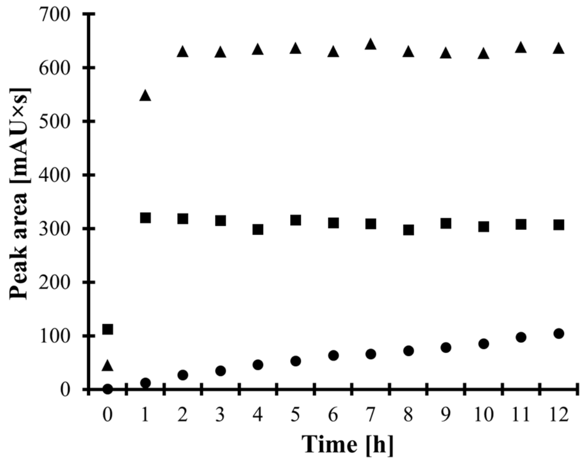

Figure S2. Reactivity of tPro toward CMQT in 0.2 mol/L PBS at pH 4.2 (dots), 6.0 (triangles) and 9.0 (squares) at room temperature as a function of time, expressed as a peak area of tPro-CMQT.

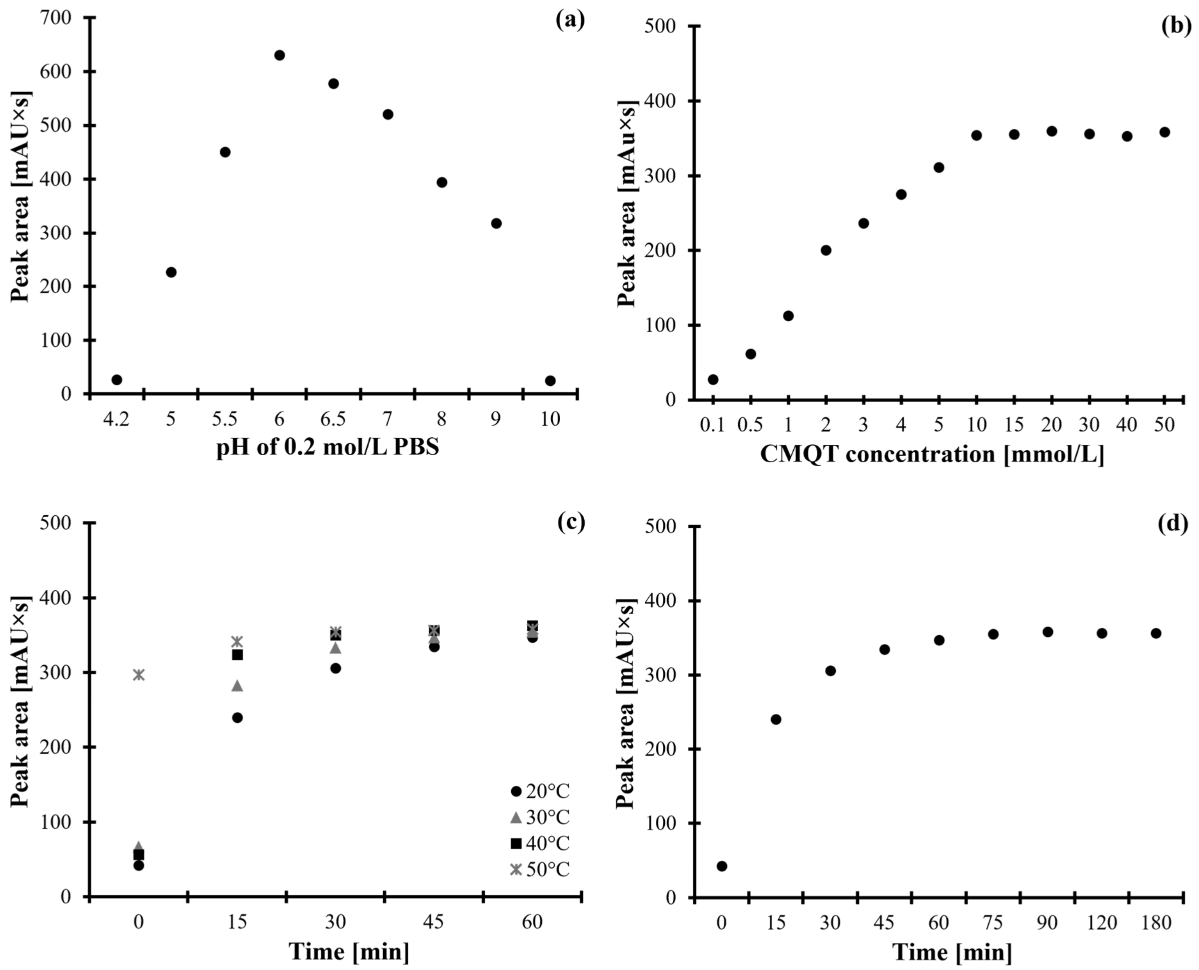

Figure S3. Derivatization reaction efficiency as a function of (a) pH of reaction medium, (b) concentration of CMQT in reaction mixture, (c) temperature, (d) time, expressed as a peak area of tPro-CMQT.

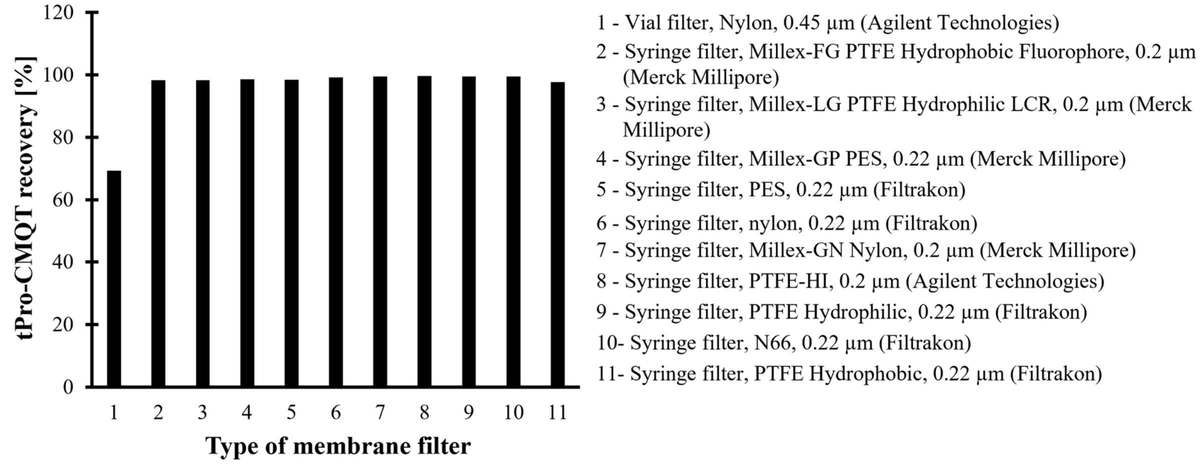

Figure S4. Membrane filtration efficiency of tPro-CMQT as a function of the type of membrane filter, expressed as a percentage of tPro-CMQT recovery.

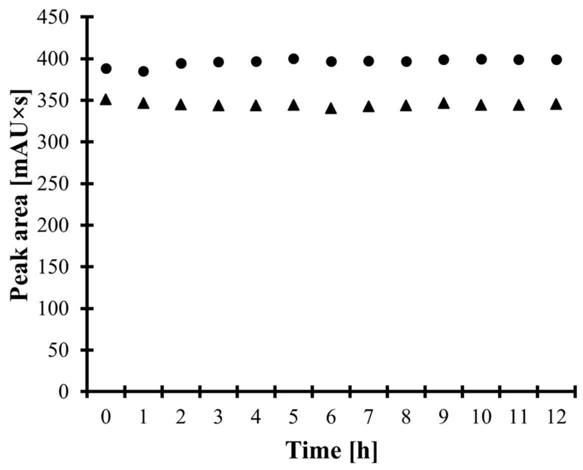

Figure S5. Examination of tPro-CMQT stability at room temperature in 0.2 mol/L PB pH 6 (dots) and samples treated with 3 mol/L PCA (triangles) as a function of time, expressed as a peak area of tPro-CMQT.

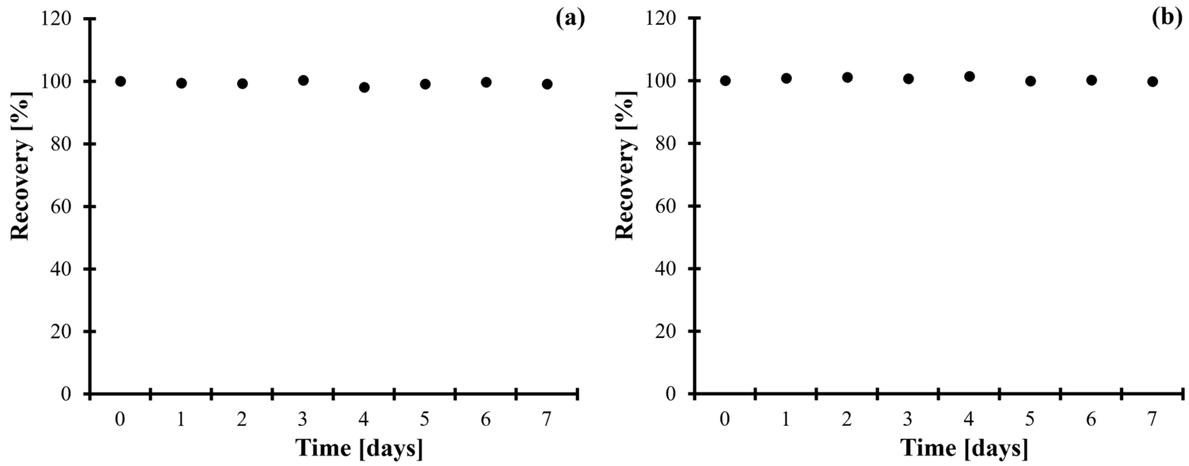

Figure S6. Examination of tPro-CMQT stability at (a) room temperature and (b) 4 °C as a function of time, expressed as the percentage of the tPro-CMQT recovery.

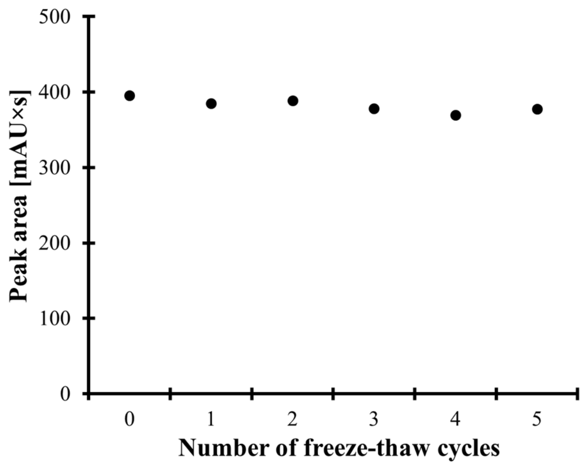

Figure S7. Freeze-thaw stability of tPro-CMQT in processed samples, expressed as a peak area of tPro-CMQT.

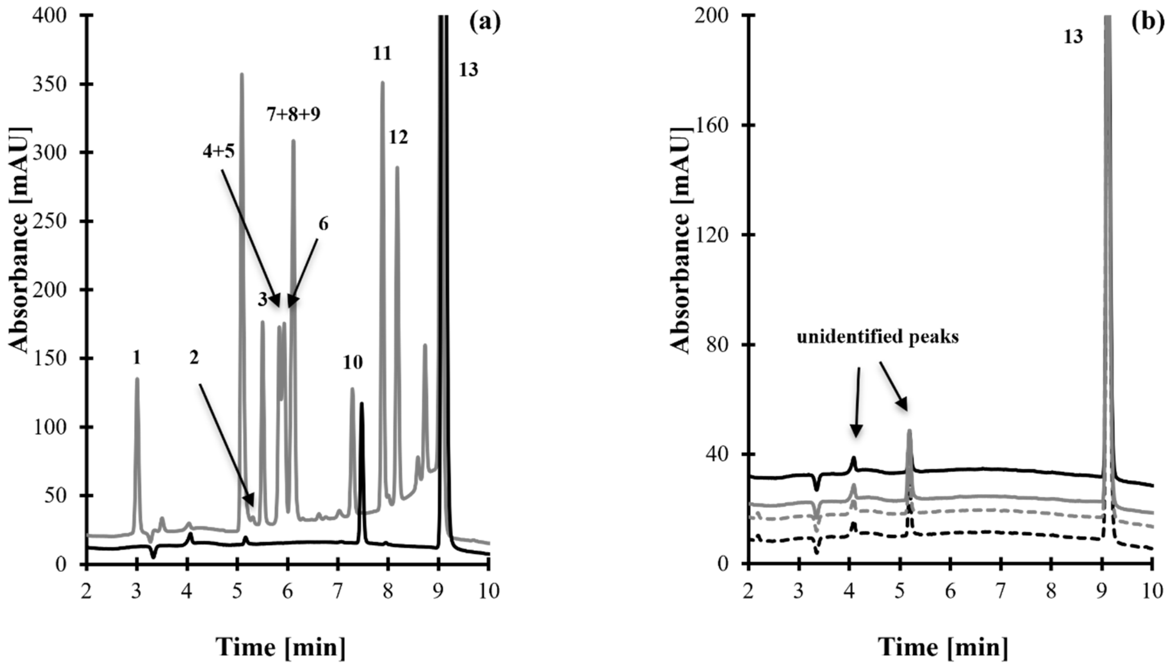

Figure S8. Representative chromatograms of standard solutions prepared according to the procedure described in section 3.5. Chromatographic conditions were as described in section 3.6. (a) Standard solution of tPro (black line) (100 µmol/L) and standard solution of HPPTCA, Cys, Glu, Hcy, Cys-Gly, N-acetyl-cysteine, captopril, mesna, cysteamine, tiopronin, methimazole, and γ-glutamyl-cysteine (grey line) (100 µmol/L). Under these conditions, their corresponding 2-*S*-quinolinium derivatives elute in the following order: 1-mesna, 2-HPPTCA, 3-N-acetyl-cysteine, 4-Glu, 5-γ-glutamyl-cysteine, 6-tiopronin, 7-methimazole, 8-Hcy, 9-cysteamine, 10-captopril, 11-Cys, 12-Cys-Gly, 13-CMQT. (b) Pharmaceutical tablet (black solid line), face cream (grey line), face foundation (black dashed line) and face serum (grey

dashed line) without tPro.

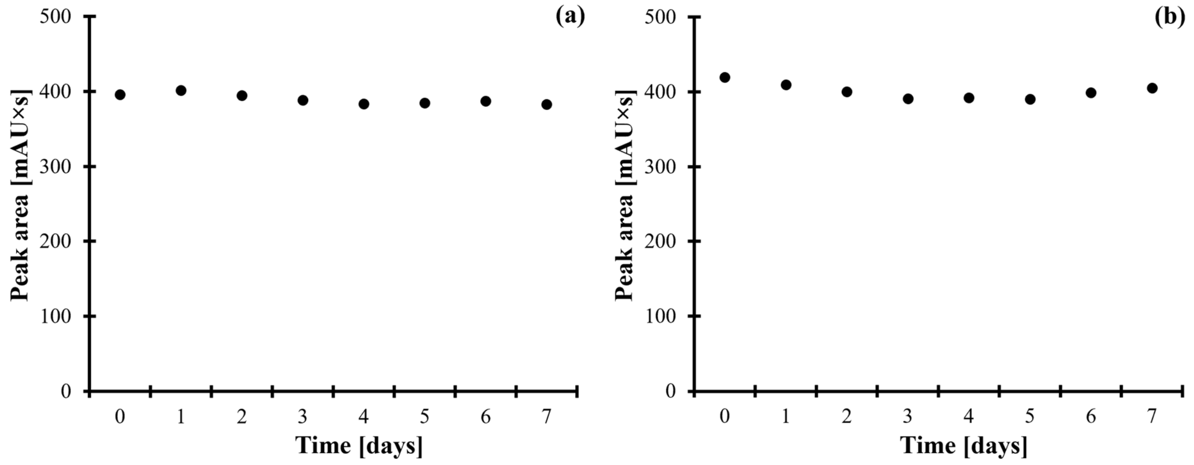

Figure S9. Examination of stability of stock solution of tPro at (a) room temperature and (b) 4 °C as a function of time, expressed as a peak area of tPro-CMQT.

Supplementary information on validation process of the presented HPLC-UV assay

The method was validated following the latest International Council for Harmonization guideline for bioanalytical methods validation in terms of selectivity, linearity, intra-/inter-assay precision and accuracy, LOQ, carry-over effect, matrix effect, and reinjection reproducibility. Moreover, parameters such as system suitability and recovery were evaluated. Samples were prepared as described below.

System suitability

In 2 mL PP microtube, powdered pharmaceutical tablet (~0.003 g) or face care product (~0.3 g) without tPro was spiked with 5, 50 or 100 µL of 0.1 mol/L tPro and filled up to 1 mL with 0.2 mol/L PBS at pH 6.0. Then samples were assayed according to the procedures described in section 3.5 and 3.6 as well as section 2.3.1.

Selectivity

Four types of samples were prepared in order to evaluate selectivity of the method. They included 1) blank standard solution, 2) standard solution of HPPTCA and reduced forms of Cys, Glu, Hcy, Cys-Gly, N-acetyl-cysteine, captopril, mesna, cysteamine, tiopronin, methimazole, and γ-glutamyl-cysteine, 3) pharmaceutical tablets and face care products (creams/foundations/sera) without tPro, 4) pharmaceutical and cosmetic products containing tPro. Samples were prepared as follows:

- **Blank standard solution sample**. In 1st hydrolytic class glass HPLC vial, 940 µL of 0.2 mol/L PBS at pH 6.0 and 60 µL of 0.25 mol/L CMQT were mixed. The sample was put aside for 60 min at room temperature and then analyzed according to procedures described in section 3.6 and 2.3.2.
- **Standard solution of low molecular mass sulfur-containing compounds, other than tPro**. In 1st hydrolytic class glass HPLC vial, 910 µL of 0.2 mol/L PBS at pH 6.0 was mixed with 60 µL of 0.25 mol/L CMQT, and then 10 µL of 0.01 mol/L mixture containing HPPTCA, reduced forms of Cys, Glu, Hcy, Cys-Gly, N-acetyl-cysteine, captopril, mesna, cysteamine, tiopronin, methimazole, and γ-glutamyl-cysteine was added. The sample was put aside for 5 min at room temperature. Thereafter, 20 µL of 3 mol/L was added. The sample was analyzed according to procedures described in section 3.6 and 2.3.2 without delay.

- **Pharmaceutical tablets and face care products without tPro**. In 2 mL PP microtube, powdered pharmaceutical tablet (~0.003 g) or face care product (~0.3 g) without tPro was mixed with 1 mL of 0.2 mol/L PBS at pH 6.0. Then sample was assayed according to the procedures described in section 3.5 and 3.6 as well as section 2.3.2.
- **Pharmaceutical and cosmetic products containing tPro**. In 2 mL PP microtube, powdered pharmaceutical tablet (~0.003 g) or face care product (~0.3 g) containing tPro was mixed with 1 mL of 0.2 mol/L PBS at pH 6.0. Then sample was assayed according to the procedures described in section 3.5 and 3.6 as well as section 2.3.2.

Linearity

In 2 mL PP microtube, powdered pharmaceutical tablet (~0.003 g) or face care product (~0.3 g) without tPro was spiked with 0.5, 1, 5, 10, 25, 50, 75, 100 or 125 µL of 0.1 mol/L tPro and filled up to 1 mL with 0.2 mol/L PBS at pH 6.0. Then samples were assayed according to the procedures described in section 3.5 and 3.6 as well as section 2.3.3.

Precision and accuracy

In 2 mL PP microtube, powdered pharmaceutical tablet (~0.003 g) or face care product (~0.3 g) without tPro was spiked with 0.5, 10, 50 or 100 µL of 0.1 mol/L tPro and filled up to 1 mL with 0.2 mol/L PBS at pH 6.0. Then samples were assayed according to the procedures described in section 3.5 and 3.6 as well as section 2.3.4.

The limit of detection

In 2 mL PP microtube, powdered pharmaceutical tablet (~0.003 g) or face care product (~0.3 g) without tPro was spiked with 0.5 µL of 0.1 mol/L tPro and filled up to 1 mL with 0.2 mol/L PBS at pH 6.0. Then samples were assayed according to the procedures described in section 3.5 and 3.6 as well as section 2.3.5.

Carry over effect

Two types of samples were prepared in order to evaluate carry over effect. They included 1) blank standard solution sample and 2) calibrator at the upper LOQ (125 µmol/L). Samples were prepared as follows:

- **Blank standard solution sample**. In 1st hydrolytic class glass HPLC vial, 940 µL of 0.2 mol/L PBS at pH 6.0 and 60 µL of 0.25 mol/L CMQT were mixed. The sample was

put aside for 60 min at room temperature and then analyzed according to procedures described in section 3.6 and 2.3.6.

- **Calibrator at the upper LOQ**. In 2 mL PP microtube, powdered pharmaceutical tablet (~0.003 g) or face care product (~0.3 g) without tPro was spiked with 125 µL of 0.1 mol/L tPro and filled up to 1 mL with 0.2 mol/L PBS at pH 6.0. Then sample was assayed according to the procedures described in section 3.5 and 3.6 as well as section 2.3.6.

Matrix effect

In 2 mL PP microtube, powdered pharmaceutical tablet (~0.003 g) or face care product (~0.3 g) without tPro was spiked with 0.5, 1, 5, 10, 25, 50, 75, 100 or 125 µL of 0.1 mol/L tPro and filled up to 1 mL with 0.2 mol/L PBS at pH 6.0. Then samples were assayed according to the procedures described in section 3.5 and 3.6 as well as section 2.3.7.

Reinjection reproducibility

In 2 mL PP microtube, powdered pharmaceutical tablet (~0.003 g) or face care product (~0.3 g) without tPro was spiked with 5, 50 or 100 µL of 0.1 mol/L tPro and filled up to 1 mL with 0.2 mol/L PBS at pH 6.0. Then samples were assayed according to the procedures described in section 3.5 and 3.6 as well as section 2.3.9.

Extraction efficiency

Two types of samples were prepared in order to evaluate extraction efficiency. Samples were prepared as follows:

- **Type 1.** In 2 mL PP microtube, powdered pharmaceutical tablet (~0.003 g) or face care product (~0.3 g) containing known amount of tPro was spiked with 5, 50 or 100 µL of 0.1 mol/L tPro and filled up to 1 mL with 0.2 mol/L PBS at pH 6.0. Then samples were extracted by vortexing at 2000 rpm for 5 min at 25 °C. Afterwards, 5, 50 or 100 µL of 0.2 mol/L PBS at pH 6.0 was added to the resulting mixture. In the next step, 10 µL of obtained supernatant was transferred into 1st hydrolytic class glass HPLC vial containing 930 µL of 0.2 mol/L PBS at pH 6.0 and 60 µL of 0.25 mol/L CMQT. The reaction mixture was put aside for 60 min at room temperature. An 5 µL aliquot of the resulting solution was introduced into HPLC-UV system and assayed according to the procedures described in section 3.6 and 2.3.9.

- **Type 2.** In 2 mL PP microtube, powdered pharmaceutical tablet (~0.003 g) or face care product (~0.3 g) containing known amount of tPro was mixed with 1 mL of 0.2 mol/L PBS at pH 6.0. Then samples were extracted by vortexing at 2000 rpm for 5 min at 25 °C. Afterwards, 5, 50 or 100 µL of 0.1 mol/L tPro was added to the resulting mixture. In the next step, 10 µL of obtained supernatant was transferred into 1st hydrolytic class glass HPLC vial containing 930 µL of 0.2 mol/L PBS at pH 6.0 and 60 µL of 0.25 mol/L CMQT. The reaction mixture was put aside for 60 min at room temperature. An 5 µL aliquot of the resulting solution was introduced into HPLC-UV system and assayed according to the procedures described in section 3.6 and 2.3.9.
